# Pain neurophysiology knowledge among physical therapy students in Saudi Arabia: a cross-sectional study

**DOI:** 10.1186/s12909-018-1329-5

**Published:** 2018-10-03

**Authors:** Faris Alodaibi, Ahmed Alhowimel, Hana Alsobayel

**Affiliations:** 1 College of Applied Medical Sciences, Health Rehabilitation Sciences, King Saud University, Riyadh, Saudi Arabia; 2 Physical Therapy and Rehabilitation Department, Prince Sattam Bin Abdul-Aziz University, Al-Kharj, Saudi Arabia

**Keywords:** Pain, Neurophysiology knowledge, Pain education, Physical therapy

## Abstract

**Background:**

Pain is a leading cause of disability and accounts for many hospital and physical therapy visits. Current pain science understanding has evolved and changed substantially in the past 20 years; however, university health science curricula may not have progressed at the same rate. This study aimed to examine knowledge about pain neurophysiology among physical therapy students in Saudi Arabia, and to compare their knowledge across different education levels and by gender.

**Methods:**

A cross-sectional study conducted to examine the pain neurophysiology knowledge among college physical therapy students in Saudi Arabia. The Revised Neurophysiology of Pain Questionnaire (12 items) was used. Descriptive statistics including frequencies and percentages were used to describe the sample. Analysis of variance and t-test were also used to examine the significant differences between scores.

**Results:**

Physical therapy students (*n* = 202) from 18 different universities in Saudi Arabia participated in this study. The mean score of the participants on the questionnaire was 6.20 ± 2.07 (i.e., 52% ± 17%) and there was no significance difference between males and females. There was a statistically significant incremental increase in total score through the educational process (*P* < 0.05); however, this increase was very small comparing early- and final educational-level students (8% in RNPQ).

**Conclusion:**

While final year physical therapy students showed higher levels of pain science knowledge than those at the beginning of their course, the magnitude of the difference was small and likely of little meaningful relevance. This may reflect the need for more emphasis on pain science in the physical therapy curriculum in Saudi Arabia.

**Electronic supplementary material:**

The online version of this article (10.1186/s12909-018-1329-5) contains supplementary material, which is available to authorized users.

## Background

Musculoskeletal pain is one of the most common complaints seen in physical therapy (PT) practice worldwide. More than a quarter of adults in the United States are expected to have complained of back pain within the last 3 months [1]. Shoulder pain and neck pain each affect one-fifth of the Dutch adult population [2]. Back pain and neck pain are estimated to be the third most important condition in terms of health care spending in the United States [3]. Although there are few studies on the prevalence of pain disorders in Saudi Arabia, a study that was conducted in a University hospital show that back pain and neck pain are one of the most prevalent conditions [4]. Inappropriate pain management leads to chronic pain and disability in some individuals [5]. Pain symptoms and experiences are complex and multidimensional [6].

Pain science has developed rapidly in the past 50 years [7, 8]. However, changes in knowledge about pain have not been covered sufficiently in many health sciences curricula [9–14]. Adillón et al. found that the knowledge of final-year PT students about pain neurophysiology was greater than that of their peers from a medical school in Spain, but this knowledge was not sufficient to deal with chronic pain conditions [15]. Integrating the International Association for the Study of Pain (IASP) curriculum into undergraduate health care programs has been shown to improve outcomes in knowledge and beliefs about pain [14]. Knowledge about pain neuroscience is crucial to guide appropriate patient management [15–17]. Even a brief educational session on pain neuroscience can result in a positive change on PT students’ pain knowledge and potentially positive attitudes and beliefs change towered patients with chronic pain [18–20].

The most common entry-level PT program in Saudi Arabia is the 5 to 6 years bachelor degree, which can be taken in about 8 to 9 semesters (plus the internship year) [21]. To our knowledge, PT undergraduate programs in Saudi Arabia do not include a stand-alone course on pain science, but pain subjects are integrated within other courses [21]. Little is known about the extent of knowledge of Saudi Arabian PT students about pain neurophysiology. Therefore, this cross-sectional study aimed to assess the pain neurophysiology knowledge of PT students in Saudi Arabia and to compare the differences in knowledge in terms of level of study and gender.

## Methods

This was a cross-sectional study to evaluate student knowledge about pain using the Revised Neurophysiology of Pain Questionnaire (RNPQ) [22, 23] across different PT schools in Saudi Arabia. The Institutional Review Board of the College of Applied Medical Sciences, King Saud University approved this study. Male and female PT students who were studying in Saudi Arabian universities were invited to participate in this study. The questionnaire was web-based and distributed through different social media websites and by direct invitation through PT student clubs. The questionnaire was administered from Oct 23rd 2016 to Dec 20th 2016. Consenting students were included in the study and their demographic data (i.e. age and gender), year of study, and school name were gathered. Questions were also asked about knowledge sources on pain (according to student perceptions). After completing this section, the student was transferred to answer the 12 items in the RNPQ.

### RNPQ

This questionnaire is composed of 12 statements on pain neurophysiology that can be answered with “true”, “false”, or “undecided” (if the question or the answer is not clear). Each item with a correct answer scores one point, with 12 points possible in total (“undecided” selection granted 0 points for any item in the questionnaire). The items in the questionnaire were originally chosen from postgraduate pain medicine examinations, and it contains items that assess individual concepts of pain biology and neurophysiology based on current pain science [22]. The questionnaire was reanalyzed recently with some poorly functioning items excluded [23]. The 12 items used (Additional file 1: Table S1) have acceptable psychometric properties and was found to be a useful assessment tool of an individual’s conceptualization of biological pain mechanisms [23]. We used the English language version of the questionnaire since the main language used in most of the curriculum courses in Saudi Arabian PT schools is English. Students were asked to identify the sources of their pain neurophysiology knowledge from five options (knowledge before university, courses taken in the PT program, article/textbook reading, internet, and others). Students were able to choose more than one option.

### Analysis

Statistical Package for the Social Sciences software (version 23) was used to analyze the data. Descriptive analysis including means with standard deviations (SD), frequencies and percentages was used to describe the sample. Analysis of variance (one-way ANOVA) and *t*-tests (independent samples) were used to analyze significant difference between scores. The scores were compared between different levels of study and between males and females. Usually males and females study same PT curriculum but in different campuses and this is the reason gender differences comparison was added to the aims of this study. *P*-values < 0.05 (two-sided) were considered significant.

## Results

A total of 202 PT students from 18 different universities and colleges in Saudi Arabia participated in the survey. About half of the respondents were female (*n* = 109, 54%) and 45% of the respondents were from King Saud University (participants’ institutions with percentage participation from each institution are listed in Additional file 2: Table S2). The mean age of the respondents was 22.4 years (SD ± 2.6).

The sample was divided into three groups: early stage of the PT program, which included PT students at the beginning of their program until the fifth semester (*n* = 58; 29%); middle level of the PT program, which included students from the sixth to the ninth semesters, inclusive (*n* = 73; 36%); and final level of the PT program, which included students in their internship year and in the early years of professional practice (*n* = 71; 35%). Physical therapists in the early years of the profession (10% of the sample) were included in the “final level” stage since their experience after graduation was minimal (average age = 25.4 ± 2.87 years).

The average score of the entire sample on the pain neurophysiology knowledge questionnaire was 6.20 ± 2.07 (i.e., 52% ± 17%). There was no significant difference between students’ average score from King Saud University ($\overline{x}$ =6.44, SD = 2.15) and Students from other schools ($\overline{x}$ =6.00, SD = 2.00); t(199) = 1.5, *p* = 0.14.There was a slight increase in the average as the students became more experienced. Between-group mean differences were significant [F (2, 199) = 3.34, p = 0,037]; however, the difference between early level PT and final level PT average scores was minimal (Table 1). The difference score average between the early level and the final level of PT program was 0.92 point (8%).

**Table 1:** Score means comparing males and females in the three different PT stages

PT level groups	Gender	N	Mean score (percentage)	SD
Early level of the PT program	Male	21	6.1 (51%)	1.6
	Female	37	5.5 (46%)	2.0
Middle level of the PT program	Male	44	6.1 (51%)	1.8
	Female	29	6.0 (50%)	2.1
Final level of the PT program	Male	28	6.4 (53%)	2.3
	Female	43	6.8 (57%)	2.4

To examine the gender difference in pain neurophysiology knowledge, we ran an independent samples *t*-test to compare the scores. There was no statistically significant difference between the average scores of males ($\overline{x}$ = 6.20, *SD* = 1.9) and females ($\overline{x}$ = 6.19, *SD* = 2.3) in the questionnaire (t(200) = − 0.04, *p* = 0.97).

**Fig. 1:**
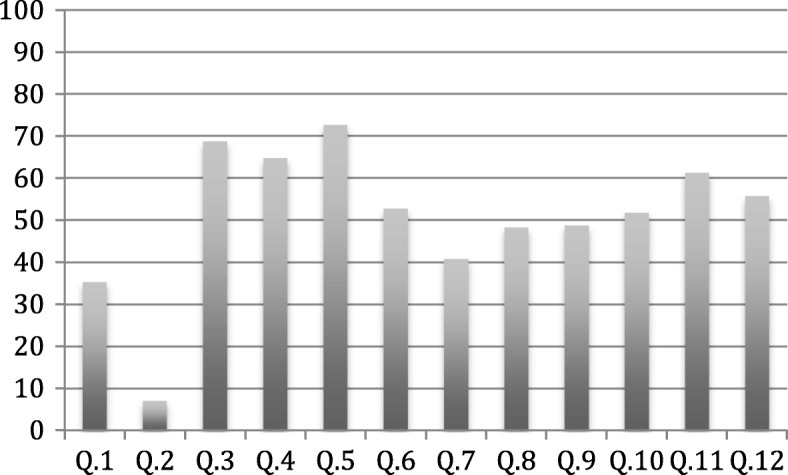
Percentage of correct answers to each of the 12 items on the questionnaire (x-axis: item number; y-axis: percentage of correct answer)

**Fig. 2:**
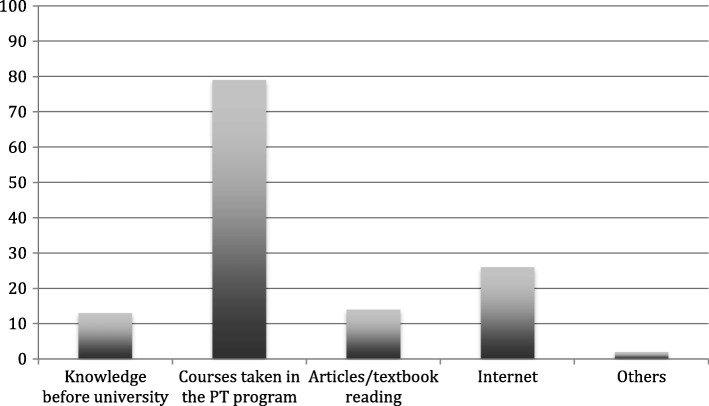
Pain neurophysiology knowledge sources (x-axis: knowledge sources; y-axis: percentages)

The percentage of correct responses to each question was analyzed to identify the most problematic items (Fig. 1). Five questions had < 50% correct answers (Items 1, 2, 7, 8 and 9). Students listed the sources of their pain neurophysiology knowledge from five options (knowledge before university, courses taken in the PT program, article/textbook reading, internet, and others). Figure 2 shows their selections (as percentages).

## Discussion

The aim of this study was to assess the pain neurophysiology knowledge of PT students in Saudi Arabia and to compare differences in their knowledge in terms of level of study and gender. The results of this study showed limited knowledge with regards to pain neurophysiology among PT students. Although there was a statistically significant difference in knowledge between early- and late-stage PT study, the increasing in understanding was minimal with progression through the PT program (only 8% difference between the early and the final level of PT program). There was no significant gender difference in knowledge. Alshami et al. [24] have shown that PT students had more negative attitudes toward chronic pain patients compared to Brazilian and Australian PT students. This might be the result of a limited knowledge in pain neuroscience. Our results of a limited knowledge were similar to other studies that show limited pain science knowledge of PT students (about 50% on the RNPQ) before being exposed to pain neuroscience education sessions [19, 20]. These studies show improvement in pain science knowledge and potentially positive attitudes and beliefs change after brief pain neuroscience education [19, 20].

Adillón et al. [15]. showed that PT students scored higher in assessment of their pain neurophysiology knowledge when compared with medicine and nutrition students, and this might suggest the same limitations are present in pain education across healthcare curricula. Comparing our results with those for students from Spain [15], there was improvement in knowledge with progression through education but the improvement was less in our sample (26% improvement in Adillón et al. compared to 8% in our study).

Correct answer percentages varied across the sample, but items with a low rate of correct answers were reviewed. For example, Item 2, “When part of your body is injured, special pain receptors convey the pain message to your brain”, and Item 1, “It is possible to have pain and not know about it”, had the lowest levels of correct answers (7% and 35%, respectively). Modern neuroscience education discourages using classical terms (i.e., pain receptors, pain fibers, and pain pathway) since such terms may convey the message that pain is an input coming from tissue damage to the brain. Therefore, it is recommended not to confuse “pain” with “nociception” because each can occur without the other [25]. Unfortunately, the classical terms might still be used in PT teaching programs in Saudi Arabia.

Item 7, “Chronic pain means that an injury hasn’t healed properly”, and Item 8, “Worse injuries always result in worse pain”, also got low rates of correct answers (41% and 48%, respectively). Incorrect answers to these two items reflect the belief of many students in the association between pain severity and persistence and tissue damage, ignoring other factors. This belief might be reflected in their approaches to delivering patient treatment in future practice, particularly adoption of the biomedical approach, which focuses purely on biological factors but discount other psychological, social, and environmental factors. These findings are consistent with a qualitative study of healthcare practitioners that suggested reliance on the biomedical approach is the general practice when dealing with back pain [26].

Our study findings highlight that the main source of knowledge about neurophysiology of the sampled students was education in the university PT program. There is evidence supporting that the inclusion of 70 min of education (as a lecture) to the curriculum significantly increases the knowledge of pain science and can have a positive change on attitudes toward chronic pain patients [15, 20, 23]. Therefore, it is of high importance to incorporate the modern pain neuroscience education into the teaching of PT students in universities.

Although this is the first study on the understanding of pain neurophysiology among students in Saudi Arabia, it has some limitations. The fact that we used student clubs to distribute the survey makes it hard to estimate the response rate. The other major limitation that needs to be considered is that almost half of the respondents were from one university, thus it may not be appropriate to generalize our findings to students throughout the country. The measurement tool itself also has some limitations. There is no established cut-off value of what is a reasonable knowledge score; therefore, we rely on the percentage score for the whole sample. It should be noted that early level PT students are expected to score low; however, the acceptable variation in the score is not established.

Knowledge of the Saudi physical therapists with regard to pain neurophysiology has not yet been studied as a function of years of experience. It would be advisable to examine that in future studies and to compare the results across different health care specialties. The pain curricula in Saudi PT schools need to be developed and to be consistent with the modern pain neuroscience and the recommendations of the IASP [27].

## Conclusion

This was the first study to measure pain neurophysiology knowledge among healthcare students in Saudi Arabia. There was no significant difference in knowledge between males and females. Our findings demonstrate only a small increase in pain neurophysiology knowledge with greater time in education in Saudi PT schools. The findings suggest that pain science curricula in Saudi PT schools appears to need development so knowledge of modern pain science is increased.

## Additional files

Additional file 1: **Table S1.** The Revised Neurophysiology of Pain Questionnaire (RNPQ) 12-items. 12 statements on pain neurophysiology that can be answered with “true”, “false”, or “undecided”. (DOCX 18 kb)

Additional file 2: **Table S2.** Participants’ institutions with percentage participation from each institution. Participants’ institutions with percentage participation from each institution. (DOCX 18 kb)

## Abbreviations

IASP: The International Association for the Study of Pain
PT: Physical therapy
RNPQ: The Revised Neurophysiology of Pain Questionnaire
SD: Standard deviation
