# Supplementary material for: Pain neurophysiology knowledge among physical therapy students in Saudi Arabia: a cross-sectional study
Source: BMC Med Educ. 2018 Oct 3;18:228. doi: 10.1186/s12909-018-1329-5 (PMC6171286; doi:10.1186/s12909-018-1329-5)
Supplement: Supplementary file 1 — Table S1. The Revised Neurophysiology of Pain Questionnaire (RNPQ) 12-items. 12 statements on pain neurophysiology that can be answered with “true”, “false”, or “undecided”. (DOCX 18 kb) [file 12909_2018_1329_MOESM1_ESM.docx]

Additional file 1: Table S1: The Revised Neurophysiology of Pain Questionnaire (RNPQ) 12-items, Moseley, L. (2003) [18], Catley, M. J. et al., (2013) [19]

| 1 | It is possible to have pain and not know about it. |
| --- | --- |
| 2 | When part of your body is injured, special pain receptors convey the pain message to your brain. |
| 3 | Pain only occurs when you are injured or at risk of being injured. |
| 4 | When you are injured, special receptors convey the danger message to your spinal cord. |
| 5 | Special nerves in your spinal cord convey ‘danger’ messages to your brain. |
| 6 | Nerves adapt by increasing their resting level of excitement. |
| 7 | Chronic pain means that an injury hasn’t healed properly. |
| 8 | Worse injuries always result in worse pain. |
| 9 | Descending neurons are always inhibitory. |
| 10 | Pain occurs whenever you are injured. |
| 11 | When you injure yourself, the environment that you are in will not affect the amount of pain you experience, as long as the injury is exactly the same. |
| 12 | The brain decides when you will experience pain. |
