# Supplementary material for: Pain neurophysiology knowledge among physical therapy students in Saudi Arabia: a cross-sectional study
Source: BMC Med Educ. 2018 Oct 3;18:228. doi: 10.1186/s12909-018-1329-5 (PMC6171286; doi:10.1186/s12909-018-1329-5)
Supplement: Supplementary file 2 — Table S2. Participants’ institutions with percentage participation from each institution. Participants’ institutions with percentage participation from each institution. (DOCX 18 kb) [file 12909_2018_1329_MOESM2_ESM.docx]

Additional file 2: Table S2: Participants’ institutions with percentage participation from each institution

| 1-King Saud University | 45% |
| --- | --- |
| 2-Taif University | 7% |
| 3-Princess Nora bint Abdul Rahman University | 6% |
| 4-Umm al-Qura University | 5% |
| 5-Prince Sattam Bin Abdulaziz University | 5% |
| 6-Majmaah University | 4.5% |
| 7-King Abdulaziz University | 4% |
| 8-Qassim University | 4% |
| 9-UOD (Imam Abdulrahman Bin Faisal University) | 3.5% |
| 10-King Khalid University | 3% |
| 11-Jazan University | 2.5% |
| 12-University of Hail | 2.5% |
| 13-Najran University | 2% |
| 14-Buraydah Colleges | 1.5 |
| 15-Al Jouf University | 1% |
| 16-Taibah University | 1% |
| 17-University of Tabuk | 0.5% |
| 18-Batterjee Medical College | 0.5% |
